# Gut Microbiome and Metabolome Dynamics as Predictors of Clinical Outcomes in Hematopoietic Stem Cell Transplantation

**DOI:** 10.1002/mco2.70334

**Published:** 2025-08-18

**Authors:** Juewon Kim, Youjin Kim, Yoo Jin Lee, Hyo‐Jin Lee, Inseon Sim, SuJin Koh, Dong Ho Suh, Eun Sung Jung, Jae‐Cheol Jo

**Affiliations:** ^1^ Department of Physiology Konkuk University College of Medicine Chungju Republic of Korea; ^2^ Department of Hematology and Oncology Ulsan University Hospital College of Medicine University of Ulsan Ulsan Republic of Korea; ^3^ HEM Pharma Inc. Suwon Republic of Korea

**Keywords:** acetate, hematopoietic stem cell transplantation, metabolome, microbiome, metabolite assay, 1‐phenylethylamine

## Abstract

Hematopoietic stem cell transplantation (HSCT) profoundly disrupts the gut microbiome and metabolome, which in turn influence immune‐related complications and patient outcomes. To systematically characterize these perturbations, we performed a longitudinal analysis of fecal microbiota composition and metabolite profiles in HSCT recipients at three critical timepoints: pre‐transplant (T1), peri‐transplant (T2), and post‐transplant (T3). We observed that reduced microbial diversity at T1 and T3 was strongly associated with increased incidence of graft‐versus‐host disease (GVHD), progressive disease (PD), and decreased overall survival (OS). Metabolomic profiling revealed a significant decline in short‐chain fatty acids (SCFAs), particularly acetate, from T1 to T2, which correlated with adverse clinical outcomes including GVHD, diarrhea, PD, and lower OS. Elevated levels of uric acid at T2 were predictive of GVHD onset, while decreased 1‐phenylethylamine was linked to transplant‐associated diarrhea. Furthermore, enrichment of beneficial bacterial taxa such as Lachnospiraceae and Ruminococcaceae was associated with improved survival. Together, these findings highlight the gut microbiome–metabolome axis as a dynamic biomarker for HSCT prognosis. This integrated insight offers potential avenues for microbiota‐targeted diagnostics and interventions aimed at mitigating transplant‐related complications and improving patient survival.

## Graphical abstract

• Whole fecal analysis generated the distinct signature of the gut microbiome and metabolome for HSCT in subjects with time point among generally 59 individuals.

• Reduced microbiota diversity in period of HSCT is linked to increased complications, GVHD, and NF with PD.

• SPMs are meaningful target for perspectives, and acetate represents close relation with overall survival of HSCT patients.

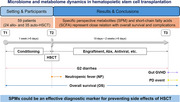

## 1 Introduction

Hematopoietic stem cell transplantation (HSCT) serves as a potent therapy against hematologic malignancies [1]. However, its broader application is hindered by complications such as graft‐versus‐host disease (GVHD), infections, and relapse [2]. Recent advancements in large‐scale DNA sequencing technologies have enabled the identification of microorganisms comprising the microbiota and their interactions with host immunity across various diseases, including cancer [3, 4]. This renewed interest highlights the importance of investigating the role of intestinal flora in patients with hematopoietic malignancies undergoing HSCT, especially in relation to clinical outcomes and transplant‐related mortality [5]. Unraveling the ecology of the microbiota and metabolites within these contexts holds promise for devising more effective strategies to mitigate associated complications. Understanding the metabolic alterations induced by HSCT can reveal underlying mechanisms of therapy‐related complications and identify potential biomarkers for predicting patient outcomes [6]. Microbiota‐derived metabolites have been shown to reduce epithelial cell damage, decrease inflammation, and alleviate GVHD in mouse models [7]. Previous studies of microbiota‐derived metabolites have primarily focused on those detected in the blood of patients undergoing allo‐HSCT [8]. Nonetheless, previous observations revealed a lack of strong correlation between metabolite concentrations in the blood and those in stool samples [9]. Moreover, most short‐chain fatty acids (SCFAs) are metabolized on first pass [10] and certain metabolites are recognized for their localized effects, particularly in maintaining intestinal barrier function and homeostasis. Furthermore, alterations in bacterial composition and metabolite levels, such as the loss of specific anaerobic commensals and reduced intestinal SCFAs, have been associated with increased severity of GVHD in patients undergoing HSCT [11]. Consequently, we directed our attention toward intestinal metabolites, leveraging targeted mass spectrometry for their precise and sensitive detection and quantification.

Early risk assessment, precise diagnosis, and prognosis rely heavily on clinical features [12]. It is crucial to develop tools for early identification and effective management of HSCT patients. A promising avenue lies in utilizing metabolomics‐based biomarkers obtained from non‐invasive biospecimens. Accordingly, we aimed to evaluate the alteration of microbiome profiles and to identify specific perspective metabolites (SPMs) in HSCT patients, elucidating the relation with complications and OS. This study provides a comprehensive investigation into the application of metabolomics and metabolome‐based biomarkers in patients undergoing HSCT.

## 2 Results

### 2.1 Participant Characteristics

Characteristics at baseline of the 58 patients with 174 fecal samples are shown in Table 1. Twenty‐three patients underwent allogeneic HSCT, while thirty‐five patients received autologous HSCT. Sixty percent of transplant recipients were male, with a median age of 61 years at the time of transplant. In the autologous HSCT cohort, 13 of the 35 patients were diagnosed with multiple myeloma, while the remaining 22 were diagnosed with lymphoma. In the allogeneic HSCT cohort, 18 out of 23 patients were diagnosed with a myeloid neoplasm. The majority of patients in this cohort received myeloablative conditioning. Additionally, a total of 26 patients who underwent allogeneic transplantation received reduced‐intensity conditioning, as detailed in Table 1.

**TABLE 1:** Patients’ information.

	Auto‐HSCT	Allo‐HSCT
Total number of patients	35	24
Median age at transplant (years)	62	61
Male sex, *n* (%)	21 (60)	14 (58.3)
Diseases, *n* (%)		
Multiple myeloma	13 (37.1)	
Lymphoma	21 (60.0)	2 (8.3)
AML	1 (2.9)	9 (37.5)
ALL		4 (16.7)
MDS		9 (37.5)
Donor source, *n* (%)		
Autologous	35 (100)	
Matched related		13 (54.2)
Matched unrelated		6 (25.0)
Haploidentical		5 (20.8)
Conditioning regimen in allo transplantation		
Flu + Bu3 + ATG		16 (66.7)
Flu + Bu2 + ATG		6 (25.0)
Flu + Mel		2 (8.3)
Pre‐transplant colonization		
VRE	6(17)	2 (8.3)
MDR GNB	1(3)	0
MRSA	0	0
HSCT−CI risk groups		
Low risk		0
Intermediate risk		5 (20.8)
High risk		19 (79.2)
GvHD prophylaxis, *n* (%)		
CsA + MTX		16 (66.7)
CsA + MTX + MMF		2 (8.3)
Steroid treatment		
< 1 mg/kg/day		11 (45.8)
1 mg/kg/day		7 (29.2)
2 mg/kg/day		6 (25.0)
Others, enbrel and/or jakavi and/or vedoluzumab		4 (16.7)
Acute GVHD grade 2–4 outcomes		
Within 100 days		10 (41.7)
Within 180 days		3 (12.5)
Acute GvHD grading, *n* (%)		
none		3 (12.5)
Grade 1–2		10 (41.7)
Grade 3–4		11 (45.8)
Stage gut GVHD		
None		14 (58.3)
Stage 1–2		6 (25.0)
Stage 3–4		4 (16.7)
Stage skin GVHD		
None		4 (16.7)
Stage 1–2		15 (62.5)
Stage 3–4		5 (20.8)
Stage liver GVHD		
None		20 (83.3)
Stage 1–2		3 (12.5)
Stage 3–4		1 (4.2)
More than one transplant over time	1	4

Abbreviations: allo‐HSCT, allogeneic hematopoietic stem cell transplantation; AML, acute myeloid leukemia; auto‐HSCT, autologous hematopoietic stem cell transplantation; Bu, Busulfan; CsA, cyclosporine; Flu, fludarabine; GVHD, graft versus host disease; HSCT‐CI, hematopoietic stem cell transplantation‐specific comorbidity index; MDR GNB, multidrug‐resistant Gram‐negative bacteria; MDS, myelodysplastic syndrome; Mel, melphalan; MMF, mycophenolate mofetil; MTX, methotrexate; VRE, vancomycin‐resistant enterococci.

### 2.2 Temporal Microbiome and Metabolome Changes Across Transplant Stages

**FIGURE 1:**
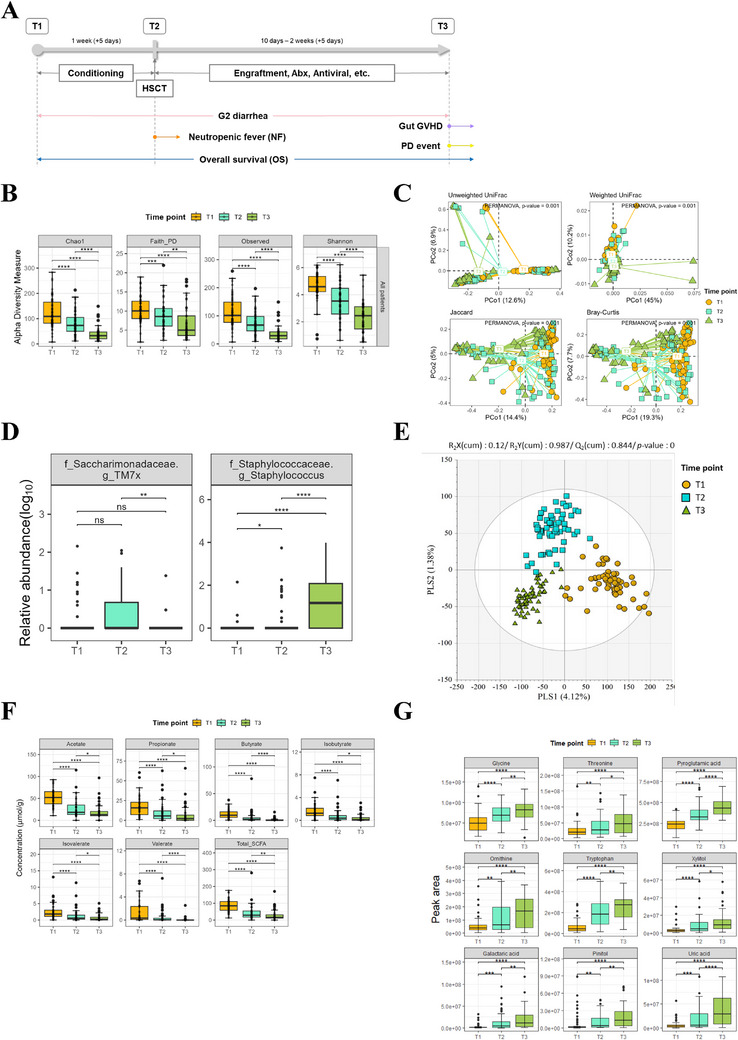
Comprehensive assessment for microbiome and metabolome of HSCT patients’ fecal samples along with time point. (**A**) Study flow diagram. (**B**) Alpha‐diversity measure of fecal microbiome for each time point, from T1 to T3. (**C**) Beta‐diversity of HSCT patients was measured with time points. (**D**) Relative abundance of gut microbiota for fecal samples that showed significance with time points, *Straphylococcus* and *Saccharimonadaceae*‐*TM7x*. (**E**) PLS‐DA analysis of whole fecal samples of patients with time point dissect. (**F**) SCFA concentration of HSCT patients along with time points. (**G**) Representative altered metabolites in overall samples along with time points. Error bars represent the mean ± s.d. *p < 0.05, **p < 0.01, ***p < 0.001, ****p < 0.0001.

Using a NGS (174 samples) and GC‐TOFMS metabolic profiling (174 samples), we categorized gut microbiota, SCFAs, and metabolites following time point with pre‐transplant (time‐point 1, T1), peri‐transplant (time‐point 2, T2), and post‐transplant (time‐point 3, T3) period (Figure 1A). We analyzed the relative abundance of bacterial genera in stool samples collected longitudinally at each time point for every participant. When comparing changes in relative abundance from the pre‐conditioning period to the peri‐engraftment period (up to Day +21 post‐transplant), a marked decrease in α‐diversity was observed, particularly immediately after transplantation (*p* < 0.0001 comparing all time points; Figure 1B). We also investigated the temporal dynamics of gut microbiota composition among patients, specifically focusing on the β‐diversity. A dissimilarity matrix was generated by calculating pairwise distances between all samples across the three distinct time points labeled as T1, T2, and T3. Our results reveal statistically significant differences in β‐diversity among the time points, indicating a meaningful variation in the microbial community structure over time (Figure 1C). During most of microbiota decreased with time point (Figure S1A–D), we sorted several bacterial taxa showed different pattern compared to other bacteria; specifically, *Staphylococcus* was elevated significantly along with time point (*p* < 0.0001, compared to T1‐T3 and T2‐T3) and *Saccharimonadaceae*‐*TM7x* was increased specifically in T2 (*p* < 0.01, compared to T2‐T3), respectively (Figure 1D).

In metabolome analysis, we classified clearly distributed whole metabolite in samples as time point using multivariate statistical analysis was performed using partial least squares‐discriminant analysis (PLS‐DA) (Figure 1E). Among the fecal metabolites, together with recently performed studies [13, 14], a total of 57 metabolites were identified as significantly altered across the transplant period in this study, with 18 metabolites elevated and 39 decreased (variable importance in projection [VIP] > 0.7 and *p* < 0.01, Kruskal–Wallis *H* test with Bonferroni‐adjusted *p*‐value < 0.05). Abundance levels were Z‐score normalized, where red indicates increased and blue indicates decreased metabolite levels. Hierarchical clustering using Ward.D2 linkage was applied to group metabolites with similar expression patterns, revealing distinct metabolic profiles across different HSCT stages (Figure S1E). These findings were further supported by volcano plot analyses (Figure S1F and Table 2) and metabolic pathway enrichment analysis across the HSCT timepoints (Figure S1G–I). Moreover, we validated that representative SCFAs, including butyrate and valerate, were significantly decreased at each time point over time point (*p* < 0.0001, compared to T1‐T2 and T2‐T3; Figure 1F). The results of temporal metabolite profiling analysis for all patients reveal a statistically significant overall decrease in most of metabolites such as organic acids, carboxylic acids, fatty acids, lipids, carbohydrates, alcohols, amins, purines, pyrimidines, and benzenoids (Figure S1E). Remarkably, results of the sequential metabolic profiling analysis for all patients indicate significant increase in the amino acid profile glycine, threonine, pyroglutamic acid, ornithine, and tryptophan. In addition, xylitol, galactaric acid, pinitol, and uric acid were also elevated progressing over time (*p* < 0.0001, compared to T1‐T3; Figure 1G). These metabolites showed distinctive propensity in whole metabolic changes, suggesting usefulness for diagnostic and target for HSCT patients.

**TABLE 2:** Significantly altered metabolites identified through Volcano plot analysis.

	Metabolite	log_2_FC	*p*‐value	Adjusted *p*‐value
T1 vs. T2	Proline	1.063128352	3.21E‐06	7.30E‐05
	beta‐Alanine	−2.004469124	4.58E‐06	7.30E‐05
	Aminomalonic acid	1.236968539	0.000653769	0.001940219
	Tryptophan	1.616297536	2.02E‐11	1.85E‐09
	Malonic acid	−2.799243156	7.62E‐05	0.000326874
	Glutaric acid	−1.832193892	0.000354302	0.001253685
	Phloretic acid	−1.730346881	0.012639592	0.025840945
	2‐Methylbutanoic acid	−1.057697549	6.00E‐05	0.000306876
	Valeric acid	−1.411958561	0.000958699	0.002520008
	Glyceric acid	−1.191342323	5.55E‐06	7.30E‐05
	Threonic acid	1.999530921	7.12E‐05	0.000326874
	Xylitol	1.204374846	0.00156904	0.003798727
	Sorbitol	2.042508826	7.03E‐06	7.63E‐05
	Glucuronic acid	1.415193128	3.25E‐05	0.000187132
	Galactaric acid	2.24381073	0.000514218	0.001576934
	Propylene glycol	2.230940908	6.95E‐05	0.000326874
	Uric acid	1.602012907	0.000298316	0.001097803
	Uracil	−1.007389686	5.39E‐06	7.30E‐05
	Thymine	−1.003881299	1.33E‐05	9.40E‐05
	3,4‐Dihydroxyhydrocinnamic acid	−2.480533201	0.000393084	0.001339398
	Ferulic acid	−1.020942453	0.00077034	0.002179107
	Phenylacetic acid	−1.14403232	1.07E‐05	8.24E‐05
	3‐Hydroxyphenylacetic acid	−3.843413104	0.000416759	0.001369352
	4‐Hydroxyphenylacetic acid	−1.158399253	0.000501468	0.001576934
T2 vs. T3	Glutaric acid	−2.27414299	0.004736764	0.033521718
	Valeric acid	−2.295624438	0.003353109	0.025707169
	4‐(Dimethylamino)butyric acid	−1.315472121	0.000380075	0.00699338
	Putrescine	−1.83957477	8.3324332979854e‐05	0.00255528
	Uric acid	1.02077712	0.000467655	0.007170705
	Urocanic acid	−1.053226727	0.008644426	0.048773245
T1 vs. T3	Proline	1.343444754	4.41E‐11	8.11E‐10
	Threonine	1.012751117	1.77E‐05	5.82E‐05
	beta‐Alanine	−3.060748586	7.84E‐08	5.15E‐07
	3‐Aminoisobutanoic acid	−2.893279953	0.000742189	0.001422529
	Aminomalonic acid	1.398217911	2.67E‐05	7.68E‐05
	gamma‐Aminobutyric acid	−1.946792335	3.32E‐05	9.26E‐05
	Ornithine	1.494501891	8.25E‐09	7.59E‐08
	Tryptophan	1.985018467	6.12E‐20	2.82E‐18
	Glycolic acid	−1.15601395	6.88E‐12	1.58E‐10
	Methylphosphate	−1.043214219	1.17E‐06	5.13E‐06
	Malonic acid	−3.088894415	4.66E‐05	0.000122436
	Citric acid	1.630967611	0.002331609	0.004047321
	Glutaric acid	−4.106336882	9.67E‐07	4.45E‐06
	5‐Aminovaleric acid	−1.105863273	0.000135296	0.000327558
	Phloretic acid	−2.852354958	0.000354869	0.00072551
	2‐Methylbutanoic acid	−1.956094803	6.27E‐11	9.62E‐10
	Valeric acid	−3.707582998	9.31E‐08	5.71E‐07
	4‐(Dimethylamino)butyric acid	−2.85095267	0.015670313	0.024856358
	Glyceric acid	−1.147061836	2.30E‐05	7.01E‐05
	Threonic acid	2.476836624	5.62E‐08	3.98E‐07
	Xylose	−1.505849244	8.82E‐06	3.12E‐05
	Xylitol	1.787608177	6.05E‐07	2.93E‐06
	Sorbitol	2.273110261	5.16E‐07	2.86E‐06
	Glucuronic acid	1.656209617	1.51E‐06	6.03E‐06
	Gluconic acid	1.479388884	0.001461998	0.00263733
	Galactaric acid	2.858893704	5.61E‐07	2.87E‐06
	Propylene glycol	2.462925295	0.000311376	0.000666199
	Pinitol	1.379781746	0.00071732	0.001404116
	Putrescine	−2.421724361	6.55E‐09	6.69E‐08
	Cadaverine	−1.584805744	0.000151246	0.000347867
	Hypoxanthine	−1.599218593	4.93E‐09	5.67E‐08
	Xanthine	−1.228958199	1.33E‐06	5.56E‐06
	Uric acid	2.622790027	1.30E‐10	1.71E‐09
	Uracil	−1.336788065	3.00E‐08	2.30E‐07
	Thymine	−1.093464836	2.36E‐05	7.01E‐05
	3,4‐Dihydroxyhydrocinnamic acid	−3.632265501	7.00E‐05	0.000174134
	Urocanic acid	−1.200803119	0.000214504	0.000481326
	Phenylacetic acid	−2.197060257	2.11E‐13	6.48E‐12
	3‐Hydroxyphenylacetic acid	−4.514157511	0.000286671	0.000627947
	4‐Hydroxybenzoic acid	−1.111831294	2.31E‐05	7.01E‐05
	4‐Hydroxyphenylacetic acid	−1.476049459	4.98E‐05	0.000127347

This table summarizes the metabolites that exhibited significant changes between different time points (T1 vs. T2, T2 vs. T3, and T1 vs. T3) based on Volcano plot analysis. Metabolites were considered significant if they met the criteria of an adjusted *p*‐value < 0.05 and an absolute log_2_ fold change (|log_2_FC|) > 1. Positive log_2_FC values indicate an increase in metabolite levels at the later time point, whereas negative values indicate a decrease.

### 2.3 Microbiome Diversity Associate With Complications and OS

**FIGURE 2:**
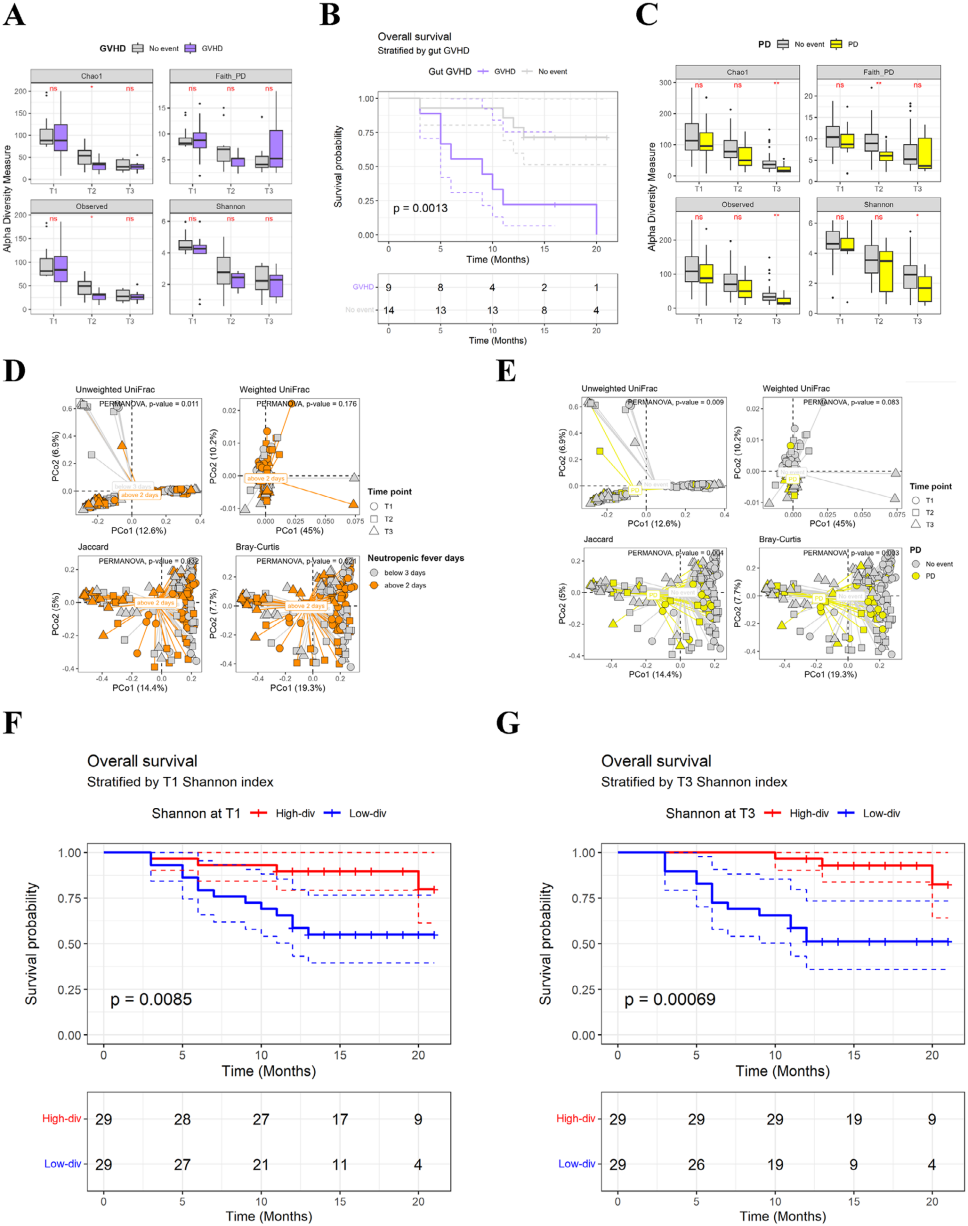
The relation of microbiota diversity with HSCT complications and OS. (**A**) α‐Diversity measure of fecal microbiome for GVHD samples (*N* = 14) compared to non‐GVHD (*N* = 9) samples. (**B**) Overall survival stratified by GVHD factor for 20 months (*p* = 0.0013). (**C**) α‐Diversity for PD (*N* = 13) versus non‐PD sample (*N* = 45). (**D**) β‐Diversity analysis of time point‐dependent samples with divided by neutropenic fever days or (**E**) PD case. (**F**) Overall survival stratified by alpha‐diversity of T1 (*p* = 0.0085) or (**G)** T3 (*p* = 0.00069). Error bars represent the mean ± SD. *p < 0.05, **p < 0.01, ***p < 0.001, ****p < 0.0001.

First, we investigated the changing aspect in microbial diversity focused on complications. Under our observation, in the group of patients who experienced GVHD, there was a notable reduction in bacterial diversity, particularly in α‐diversity, compared to those who did not experience GVHD (Figure 2A). The group without GVHD showed a significant increase in the abundance of beneficial bacterial taxa such as Lachnospiraceae, Butyricicoccaceae, and Ruminococcaceae. Conversely, the group with GVHD showed an elevated abundance of Staphylococcaceae (Figure S2A–D). And more importantly, induced GVHD was significantly associated with reduced OS for 20 months observation (*p* = 0.0013; Figure 2B). In addition, we observed a significantly decreased α‐diversity in the group that experienced PD events (*n* = 13) compared to the no‐event group (*n* = 45) (*p* < 0.05; Figure 2C). Then, we categorized group as patients with NF lasting more than 2 days or not in T2. Although the analysis of α‐diversity based on the occurrence period of NF revealed a significant decreasing trend over time, no significant differences were observed between the groups at each specific time point (Figure S2E). When comparing β‐diversity between groups based on the occurrence period of NF, including all time points (T1 + T2 + T3), a significant difference was observed (Figure 2D). Moreover, β‐diversity showed meaningful relation with PD event (Figure 2E). With consideration for major complications of HSCT, we have comprehensively examined the survival factors of HSCT patients involving microbiota diversity analysis. Our findings reveal that patients with high α‐diversity at the T1 stage exhibited significantly longer OS compared to those in the low α‐diversity group (*p* = 0.009; Figure 2F). We also observed a significantly longer in OS in patients with high α‐diversity at the T3 stage (*p* = 0.00069; Figure 2G). These findings indicate that dynamic shifts in gut microbial diversity and composition are associated with HSCT‐related complications and clinical outcomes.

### 2.4 The Serial Measurements of SCFA Have the Diagnostic Potential for HSCT Outcomes

**FIGURE 3:**
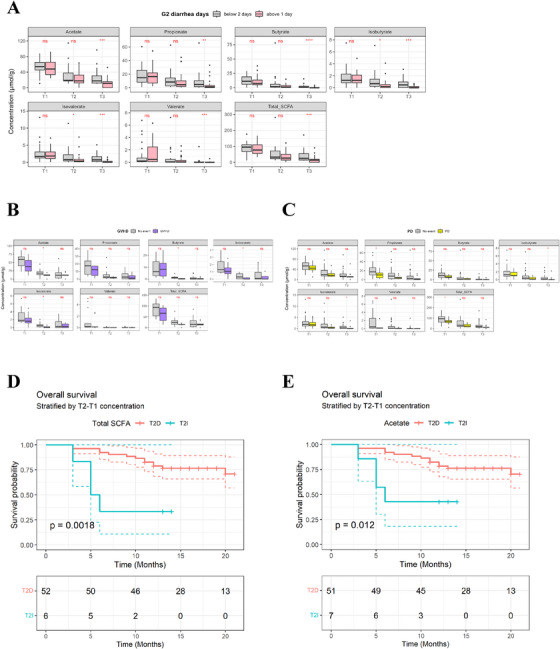
The impact of SCFA alteration with HSCT complications and OS. (**A**) Significant changes of SCFA concentration dependent on HSCT complication, G2 diarrhea days. (**B**) Significantly altered SCFA dependent on GVHD samples. (**C**) SCFA changes association with PD cases. (**D**) Overall survival piled with T2‐T1 concentrations of total SCFA (*p* = 0.0018). (**E**) Overall survival stratified with T2‐T1 acetate concentration (*p* = 0.012). T2D and T2I: T2D indicates a decrease, and T2I an increase, in metabolite concentrations at time point T2 compared to T1.

The analysis of SCFA demonstrated a significant decline in all SCFA levels over time (*p* < 0.0001, compared to T1–T3; Figure 1F). Specifically, significant reductions in SCFA in T3 were identified in patients with diarrhea lasting more than 2 days, prominent in butyrate (*p* < 0.0001; Figure 3A). The isobutyrate, acetate, valerate, and isovalerate also depicted remarkable diminution in T3 (*p* < 0.001; Figure 3A). Alternatively, the nondiarrhea group exhibited an increase in Lachnospiraceae and Ruminococcaceae, while the diarrhea group showed an increase in Staphylococcaceae and Desulfovibrionaceae (Figure S3A–D). Furthermore, the analysis of SCFA based on the presence or absence of GVHD revealed a diminishing trend in SCFA levels over time within each respective group. When comparing SCFA differences between the groups at different time points, a noteworthy reduction in acetate, butyrate, isobutyrate, and isovalerate was specifically identified during the T2 in patients with GVHD (*p* < 0.05; Figure 3B). For patients who experienced PD, total SCFA, and propionate concentration at T1, isobutyrate and isovalerate at T3 showed associations (*p* < 0.05; Figure 3C). Moreover, SCFA concentration that was satisfied by T2‐T1 showed close correlation with OS (*p* = 0.0018; Figure 3D) and more specifically, concentration of acetate displayed notable association with OS (*p* = 0.012; Figure 3E).

### 2.5 The Availability of SPM for Predictive Factor

**FIGURE 4:**
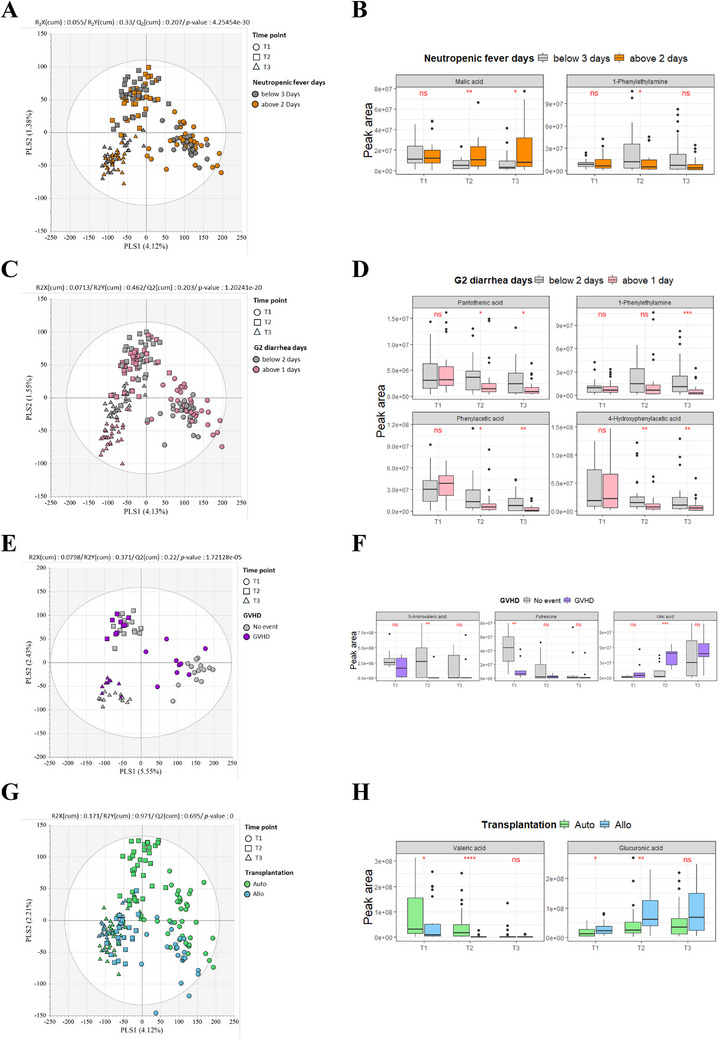
The SPMs impact on HSCT complications and transplantation types. (**A**) PLS‐DA analysis of NF factors. (**B**) Notable metabolites which shifted by NF. (**C**) PLS‐DA dissected by G2 diarrhea cases. (**D**) Significant alteration of SPMs for G2 diarrhea samples. (**E**) PLS‐DA analysis of whole fecal samples of patients with GVHD factor. (**F**) Remarkable changes of specific metabolites for GVHD patients. (**G**) PLS‐DA analysis of whole fecal samples of patients with transplant type dissect. (**H**) SPMs sorted by transplant types. Error bars represent the mean ± SD.

With significant metabolome changes using PLS‐DA model (Figure 4A), SPMs represented remarkable alteration in NF factors. Among the altered metabolites (Figure S4A), SPMs and malic acid were significantly decreased in the non‐NF group at T2 (*p* < 0.01), whereas they were markedly elevated in patients with NF at T3 (*p* < 0.05). Additionally, 1‐phenylethylamine was significantly reduced at T2 in the NF group (*p* < 0.05; Figure 4B). Moreover, for G2 diarrhea factor (Figure 4C), within altered metabolites (Figure S4B), SPMs, pantothenic acid, and phenylacetic acid described decrement chronologically in G2 diarrhea group with an inverse aspect in non‐G2 diarrhea group (*p* < 0.05; Figure 4D). The SPM 4‐hydroxyphenylacetic acid was greatly decreased in G2 diarrhea patients at T2 and T3 (*p* < 0.01; Figure 4D). Interestingly, SPM of NF factor and 1‐phenylethylamine displayed similar tendency, elevated sequentially in non‐G2 diarrhea patients with decrease in G2 diarrhea group (*p* < 0.001; Figure 4D). In case of GVHD, with apparent discrete allocation of metabolome (Figure 4D and Figure S4C), several SPMs represent notable fluctuations. The organic acid putrescine and 5‐aminovaleric acid were declined more than half (*p* < 0.05) while more dramatically, uric acid was greatly enlarged in GVHD samples at T2 (*p* < 0.001; Figure 4F). Significantly altered metabolites with complications are listed in Table 2.

### 2.6 Comparative Analysis of the Microbiome and Metabolome in Autologous Versus Allogeneic HSCT

In this study, we also investigated the implications of intestinal microbiota and metabolome in patients undergoing auto‐ or allo‐HSCT. Notably, pre‐transplant microbial α‐diversity, assessed by the Shannon index, was significantly higher in patients receiving auto‐HSCT compared to those undergoing allo‐HSCT (*p* < 0.0001; Figure 5A). Alpha diversity decreased further at post‐transplant days 14 and 28 in both the auto‐ and allo‐HSCT cohorts. However, the auto‐HSCT cohort maintained higher α‐diversity levels post‐transplant and exhibited faster recovery compared to the allo‐HSCT cohort. Significant differences in α‐diversity between the groups were observed at T1 and T2, with the most pronounced distinction at T2. Aside from levofloxacin prophylaxis, which is generally initiated immediately prior to transplant, 18% of patients in the auto‐HSCT cohort received antibiotics within 30 days before transplant, compared to 56% in the allo‐HSCT cohort. When examining β‐diversity differences between groups at different time points, significant distinctions were noted at T1 and T2 (Figure S5B). The group differences at T1 and T2, especially the prominent distinction at T2, were confirmed. Overall, these findings suggest that the early impact of transplantation on the microbiome is similar between auto‐ and allo‐HSCT recipients. We subsequently performed a differential abundance analysis to identify specific bacterial taxa that are enriched or depleted in patients post‐transplant (Figure S5C–E). The bacterial taxon Methanobacteriaceae, a commensal involved in nitrate metabolism, was enriched in the auto‐HSCT cohort. Elevated serum nitrate levels have been linked to increased risk of steroid‐refractory GVHD and post‐transplant thrombotic microangiopathy. In the allo‐HSCT cohort, we observed a significant post‐transplant decrease in the relative abundance of Prevotellaceae and Christensenellaceae, commensal bacteria known to ameliorate colitis and protect gut epithelial integrity. Additionally, we validated a significant reduction in SCFA‐metabolizing taxa—including Ruminococcaceae, Anaerofustaceae, Anaerovoracaceae, and Lachnospiraceae—in the auto‐HSCT cohort. We further examined the relative abundance of taxa involved in SCFA metabolism, which have been previously shown to influence immune homeostasis, maintain gut epithelial barrier integrity, and protect against GVHD. The results of sequential SCFA analysis for all patients indicate a statistically significant overall reduction over time from T1 to T3. Analyzing SCFA differences between groups at different time points revealed a significant decrease in SCFA levels, particularly at T2, in allo‐transplant recipients (Figure S5F). Together with clear separate distribution of metabolites in auto‐ and allo‐HSCT (Figure 4G), we discovered distinguishing feature of SPMs. Among 46 significantly changed metabolites in auto‐ versus allo‐HSCT (Figure S5G), valeric acid was greatly reduced only in allo‐HSCT samples compared to auto‐HSCT patients (*p* < 0.0001; Figure 4H). And another SPM glucuronic acid was relatively increased in allo‐HSCT patients (*p* < 0.05; Figure 4H). Additionally, overall survival (OS) differed significantly depending on the type of transplantation. As shown in Figure S5H, consistent with the differences observed in alpha diversity, OS varied significantly between transplantation methods. This suggests that the relationship between autologous transplantation and gut microbial diversity may also be linked to survival outcomes.

## 3 Discussion

In this comprehensive analysis using fecal specimens from HSCT patients, we aimed to identify distinct microbiota and associated SPMs that could serve as indicators of adverse clinical outcomes following HSCT. In this study, we uncovered the index of microbiota and metabolome in HSCT patients at specific time point, pre‐ (T1), peri‐ (T2), and post (T3)‐HSCT. The analysis suggests that microbiome diversity is strongly linked to HSCT complications such as NF, diarrhea, and GVHD, and is also associated with OS. Moreover, specific bacterial taxa and SPM have association with transplant complications. In this context from our findings, the reduction of 1‐phenylethylamine and increase in uric acid were associated with NF and G2 diarrhea and GVHD, respectively.

The intestinal microbiome undergoes profound changes during transplantation, driven by multiple transplant‐related factors such as the conditioning regimen, broad‐spectrum antibiotics, and nutrition [15]. These alterations in gut flora composition are closely associated with transplant outcomes, including OS, progression‐free survival (PFS), treatment‐related mortality (TRM), and GVHD [16]. In other vein, from the aspect of metabolomics, the comprehensive study of metabolites within a biological system has provided new insights into the complex metabolic changes that occur during and after HSCT [13, 17]. Metabolites, the small molecular intermediates and products of metabolism, are crucial indicators of physiological and pathological states [18]. They play pivotal roles in cellular signaling, energy production, and biosynthesis, influencing the success of HSCT and the patient's recovery process [19]. Microbiota‐derived metabolites, including SCFAs, tryptophan derivatives such as indoles, and secondary bile acids, exhibit immunomodulatory effects. For instance, these metabolites can modulate regulatory T‐cell function, induce type I interferon signaling, contribute to tissue homeostasis, and directly affect intestinal epithelial cells [20, 21, 22]. Through our study, we delineated specified bacterial taxa and 11 SPMs that correlated with NF duration, grade 2 diarrhea, GVHD, as well as transplantation type. There have been lots of challenges to elucidate the interactions of metabolite changes with HSCT complications and perspectives [23, 24]; in our knowledge, this is the first study to uncover altered metabolites as SPMs for the potential role of diagnostic target of complication. Especially, the correlation of acetate concentration with OS is a remarkable finding of this study. Together with performed study about relation of α‐diversity with OS [25], these identified factors may also play a role in militate for OS. We also observed a close correlation between microbial diversity and the complications of HSCT patients [26], and those with high α‐diversity in T1 demonstrated a higher OS. Our findings offer compelling evidence for the significance of microbial diversity pre‐HSCT, which may support metabolite production in humans and possibly impact HSCT outcomes. Moreover, the identified SPMs may be useful for predicting complications and OS, as they are influenced by microbial diversity and metabolism.

Our non‐interventional study focused solely on establishing associations between microbiome signatures, metabolites, and clinical outcomes. A major strength of our study is the comprehensive collection of detailed data on peri‐transplant complications combined with longitudinal serial sampling. However, we acknowledge several inherent limitations. While there are conceivable mechanisms through which SPM, such as 1‐phenylethylamine, might influence outcomes—potentially serving as biomarkers for conditions like NF and diarrhea—these mechanisms were not explored in‐depth in our study. Additionally, the diversity in underlying diseases, conditioning regimens, antibiotic exposures, and graft sources introduced considerable heterogeneity within the study population. Moreover, the small sample size may have constrained the statistical power and generalizability of our results. Although the alpha diversity of microbiome at time point T1 was predictive of OS, our study lacked the power for multivariable analysis. Therefore, these findings should be considered exploratory and require validation in a larger, international cohort. Additionally, as an observational study, our research can only show correlations and not causative relationships. Future studies using defined medical treatments with specific changes in bacterial composition and SPMs in model animals could provide an experimental system to examine the consequences of altered bacterial metabolism and its effects on HSCT complications, ultimately impacting patient outcomes and survival.

Moving forward, SPMs could be developed into an in‐hospital screening test to provide a snapshot of a clinically relevant, functional microbiome signature. Together with further preclinical investigations, our findings may pave the way for the development of novel microbiome‐based precision therapies. These therapies could utilize cocktails of microbial metabolites, which holds potential for clinical trial exploration to address conditions such as GVHD, NF, or G2 diarrhea in patients who have undergone HSCT and exhibit microbial injuries validated by biomarkers. Our study is observational, and while we have identified significant associations between gut microbiome/metabolome alterations and HSCT outcomes, causality cannot be inferred. The relatively small sample size and heterogeneity in conditioning regimens, antibiotic exposures, and graft sources may impact the generalizability of our findings. While we identified specific microbial taxa and metabolites linked to HSCT complications, further mechanistic studies are required to confirm their functional roles. Our study captures microbiome and metabolome changes at defined time points, but longer follow‐up is needed to assess their sustained impact on long‐term survival and complications.

In conclusion, alpha‐diversity and GVHD could be a precision factor for survival, more interestingly SCFA and more specifically acetate represented close correlation with OS. SPMs could be an effective diagnostic marker for preventing side effects of HSCT. Future and confirmatory studies are warranted to extend these results and certain bacterial or metabolite manipulation could reduce severity and risk of complications and OS in HSCT.

## 4 Methods

### 4.1 Population Characteristics and Sampling Procedures

This study was prospectively designed and carried out at the Hematology and Transplantation Unit of Ulsan University Hospital in Korea between March 2022 and May 2023. Stool samples were collected serially from a total of 60 patients undergoing auto‐ or allo‐HSCT with diverse conditioning regimens were included in the study. Sampling occurred three times: before the conditioning regimen, on the day of transplantation or the following day, and on day 14 post‐transplant. Two participants were excluded because of inadequate sample volumes, the analysis included 174 samples from 58 patients. The stool samples collected from the hospital were immediately stored in a deep freezer at −80°C and maintained under these conditions until analysis to ensure sample stability. The Hematopoietic Cell Transplantation Comorbidity Index (HCT‐CI) is a validated tool used to assess the risk of non‐relapse mortality following hematopoietic stem cell transplantation.

### 4.2 Definition

Acute GVHD was diagnosed clinically and confirmed by biopsy whenever feasible and graded using the modified Glucksberg criteria [27]. A diagnosis of neutropenic fever required either a single oral temperature ≥38.3°C or a continuous temperature ≥38.0°C sustained for more than 1 h, accompanied by an ANC <500/µL, or a projected decrease to below 500/µL within the next 48 h [28]. According to the Common Terminology Criteria for Adverse Events (CTCAE) Version 5.0 (November 27, 2017; U.S. Department of Health and Human Services), diarrhea was considered Grade 2 when meeting the specified criteria. Progressive disease was defined, in accordance with the European LeukemiaNet (ELN) [29] and the 2023 International Working Group (IWG) criteria [30], considered hematologic relapse, characterized by ≥5% blasts in the bone marrow, recurrence of blasts in the peripheral blood, or the appearance of extramedullary disease.

### 4.3 Antimicrobial Strategy

Prophylactic antibiotics were administered to patients with absolute neutrophil counts (ANC) <0.5 × 10⁹/L prior to hematopoietic stem cell transplantation (HSCT). Regimens included either ciprofloxacin (500 mg orally, twice daily) or cefpodoxime (100 mg orally, twice daily), which were continued until ANC exceeded 0.5 × 10⁹/L or until empiric or therapeutic antibiotics were initiated in the presence of infection. Sulfamethoxazole (400 mg) and trimethoprim (80 mg) were administered orally once daily to all patients from one month prior to bone marrow transplantation unit admission until the completion of immunosuppressive therapy. According to the institutional infection protocol, in both auto‐ and allo‐ HSCT recipients, cefepime was used as the first‐line antibiotic. Patients who remained febrile after first‐line treatment received parenteral vancomycin. The treating physician determined whether to add anaerobic or Gram‐positive coverage or to escalate to carbapenem therapy as needed. Probiotic preparations were not administered to any patient prior to transplantation.

### 4.4 GVHD Management

Cyclosporin A (CsA) and methotrexate (MTX) were administered as standard prophylaxis against acute GVHD in all allogeneic transplant recipients. Anti‐thymocyte globulin (ATG) was added to the conditioning regimen for all patients receiving allografts. GVHD prophylaxis for all patients consisted of a short course of MTX at 10–15 mg/kg/day intravenously on days +1, +3, and +6, combined with CsA administered at 1.5 mg/kg/day IV until engraftment, followed by 6 mg/kg/day orally until 180 days post‐transplant. To maintain trough levels between 150 and 250 ng/mL, CsA doses were tailored based on therapeutic drug monitoring conducted the morning after dosing. Grading of acute GVHD followed the modified Glucksberg criteria [27]. GVHD symptoms were recorded daily during hospitalization, starting after engraftment, and assessed at each outpatient visit, which were routinely scheduled at weekly intervals for 3 months. GI GVHD was confirmed by endoscopic biopsy. Treatment of grade III to IV acute GVHD involved prednisolone 2 mg/kg/day plus CsA and/or MMF, whereas grade I acute skin GVHD received topical steroid therapy. A non‐steroidal immunosuppressant, commonly a calcineurin inhibitor, was maintained to permit eventual reduction of systemic steroids. For corticosteroid‐refractory patients, second‐line therapies such as ruxolitinib, etanercept, and vedolizumab were administered per the transplant physician's discretion. Nutritional support was provided through parenteral or oral routes, while pharmacologic agents such as loperamide, dioctahedral smectite, and trimebutine were employed for symptom management. All patients did not receive probiotic preparations prior to transplantation.

### 4.5 Extraction of DNA and Next‐Generation Sequencing Analysis

Fecal bacterial genomic DNA extraction was performed using the Mag‐Bind Universal Pathogen Kit (Omega Bio‐tek, Norcross, GA, USA). Samples were resuspended in 275 µL of SLX‐Mlus Buffer and processed by bead beating with a MixerMill MM400 (Retsch, Haan, Nordrhein‐Westfalen, Germany). Further steps for DNA extraction, including isolation, cleaning, and elution procedures, followed the manufacturer's protocols. The V3‐V4 region of the 16S rRNA gene was PCR amplified from the bacterial DNA with 16S amplicon PCR primer 341F (5'‐TCGTCGGCAGCGTCAGATGTGTATAAGAGACAGCCTACGGGNGGCWGCAG‐3') and 805R (5'‐GTCTCGTGGGCTCGGAGATGTGTATAAGAGACAGGACTACHVGGGTATCTAATCC‐3'). PCR amplification was performed using 2× KAPA HiFi HotStart ReadyMix (Roche, Basel, Switzerland) with the following cycling conditions: initial denaturation at 95°C for 3 min; 25 cycles of 95°C for 30 s, annealing at 55°C for 30 s, and extension at 72°C for 30 s; followed by a final extension at 72°C for 5 min. The PCR products were purified using HiAccuBead (AccuGene, Incheon, Republic of Korea) with a magnetic stand. For library preparation, index PCR was conducted using IDT indexing primers (Integrated DNA Technologies, Coralville, IA, USA), 2× KAPA HiFi HotStart ReadyMix, and PCR‐grade water. The cycling parameters were as follows: initial denaturation at 95°C for 3 min; eight cycles of 95°C for 30 s, annealing at 55°C for 30 s, extension at 72°C for 30 s, and a final extension at 72°C for 5 min. After cleanup, library concentrations were quantified using the Qubit 4.0 fluorometer with the 1× dsDNA HS assay kit (ThermoFisher Scientific, Waltham, MA, USA). Sequencing was performed on an Illumina MiSeq platform (Illumina, San Diego, CA, USA). These all experiments were conducted at HEM Pharma Inc.

Basic microbiome bioinformatics analyses were conducted using QIIME2 version 2021.2 [31]. The raw sequencing files (fastq.gz) underwent demultiplexing and quality control via the q2‐demux plugin, and were subsequently denoised to produce amplicon sequence variants (ASVs) and removing chimeric sequences with DADA2 [32] via q2‐dada2 plugin. A phylogenetic tree was constructed using FastTree2 [33] with ASVs aligned by MAFFT [34] via q2‐phylogeny plugin. ASVs were assigned taxonomy against the Silva 138 99% reference database using classify‐sklearn from q2‐feature‐classifier [35]. Alpha diversity metrics, including the Shannon index, Faith's phylogenetic diversity, observed ASVs, and Chao1 index, were calculated using the q2‐diversity plugin. Beta diversity was assessed by principal coordinate analysis (PCoA) using weighted UniFrac distances, unweighted UniFrac, Jaccard, and Bray‐Curtis distance with phyloseq v1.46.0 R package [36]. For alpha‐ and beta‐diversity analyses, ASV data were rarefied (subsampled without replacement) to 8000 sequences per sample. The relative abundance of each taxon was expressed as the log‐transformed rarefied counts, with a pseudo‐count of 1 added to each value to avoid logarithm of zero.

### 4.6 Sample Preparation for Metabolomics and GC‐TOF‐MS Analysis

Approximately 50 mg of dried stool sample was placed into a 2‐mL microcentrifuge tube containing 1 mL of 50% methanol, with ceramic beads added to aid the extraction process. For extraction, the samples were vortexed for 10 min and subsequently sonicated for 10 min at room temperature. The samples were then centrifuged at 13,000 rpm for 10 min at 4°C, and the supernatant was filtered through a 0.2‐µm polytetrafluoroethylene syringe filter. The filtered supernatants were completely dried using a speed vacuum concentrator (Labogene, Daejeon, Korea). The dried samples were derivatized for GC‐TOF‐MS analysis by first adding 40 µL of methoxyamine hydrochloride in pyridine (20 mg/mL) and incubating at 30°C for 120 min, followed by the addition of 40 µL of N‐methyl‐N‐(trimethylsilyl) trifluoroacetamide (MSTFA) and incubation at 37°C for 60 min. Metabolic profiling of stool samples was carried out using an Agilent 7890 gas chromatograph (Agilent Technologies, Palo Alto, CA, USA) equipped with a PAL‐3 autosampler (CTC Analytics AG, Zwingen, Switzerland) and linked to a Pegasus BT time‐of‐flight mass spectrometer (TOF‐MS) system (LECO Corp., St. Joseph, MI, USA). One microliter of the derivatized sample was injected onto an Rtx‐5MS column (30 m × 0.25 mm, 0.25 µm film thickness; Restek Corp., Bellefonte, PA, USA) using helium as the carrier gas at a constant flow rate of 1.0 mL/min. The injection was performed with a split ratio of 10:1. The oven temperature was held at 75°C for 2 min, then ramped at 15°C per minute to 300°C, where it was held for an additional 3 min. The front inlet and transfer line temperatures were both maintained at 250°C. Electron ionization was carried out at −70 eV, and mass spectrometry data were acquired using a full scan range of 45–600 *m*/*z*. Raw data obtained from GC‐TOF‐MS were initially processed using LECO Chroma TOF software (version 5.40, LECO Corp.) and subsequently converted to NetCDF format (*.cdf) using the same software. After conversion, peak identification, retention time correction, and data alignment were performed using the Metalign software suite (http://www.metalign.nl). We conducted advanced multivariate statistical analyses—including principal component analysis (PCA), partial least squares‐discriminant analysis (PLS‐DA), and orthogonal PLS‐DA (OPLS‐DA)—using SIMCA P+ software (version 16.0, Umetrics, Umeå, Sweden). A heat map was generated to visualize the relative concentrations of discriminant metabolites, with fold changes normalized to the mean value of each metabolite across all samples. This allowed for comparative assessment of metabolic patterns across different transplant stages or clinical outcomes.

### 4.7 SCFAs Analysis

SCFAs were extracted by suspending 0.2 g of fecal material in 1 mL of deionized water (dH_2_O). The suspension was thoroughly vortexed and then centrifuged at 13,000 rpm for 10 min at 4°C to separate the supernatant for further analysis. A 150 µL aliquot of the supernatant was collected and transferred into a 10‐mL screw‐cap vial containing 150 µL of buffer solution composed of ammonium sulfate ((NH_4_)_2_SO_4_) and sodium dihydrogen phosphate (NaH_2_PO_4_), with 2‐ethylbutyric acid included as an internal standard [37]. SCFAs were analyzed using a headspace sampler‐gas chromatography system with a flame ionization detector (HSS‐GC‐FID), comprising an Agilent 7890B GC system coupled with a 7697A headspace sampler and FID detector (Agilent Technologies, USA). An HP‐innowax capillary column (30 m × 0.32 mm i.d. × 0.50 µm film thickness; Agilent Technologies) was employed, with nitrogen used as the carrier gas at a constant flow rate. The operating conditions were set as follows: oven temperature at 85°C, loop temperature at 90°C, transfer line temperature at 100°C, and FID temperature at 250°C. The column temperature was programmed to start at 60°C, then increase to 140°C at a rate of 30°C/min, further rise to 170°C at 30°C/min, and finally reach 180°C at 40°C/min, where it was held for 0.75 min. Data acquisition and processing were performed using ChemStation software (Agilent Technologies). Identification and quantification of SCFAs were achieved by comparison with authentic standard compounds.

### 4.8 Statistical Analysis

All statistical analyses were conducted using R software. To evaluate differences between two independent groups at a given time point, the Wilcoxon rank‐sum test was employed to determine statistical significance. Additionally, the Wilcoxon signed‐rank test was applied to analyze paired data within the same group across different time points. For comparisons involving three or more related groups, the Friedman test was used to assess overall significance, followed by pairwise comparisons using the Wilcoxon signed‐rank test. In the graphical results, only pairwise *p*‐values are displayed, and these were adjusted using the Bonferroni correction method. To assess the significance of beta‐diversity, permutational multivariate analysis of variance (PERMANOVA) was performed using the “adonis2” function with 999 permutations, implemented in the vegan v2.6.4 package in R. For microbial taxonomic profiling analysis, DESeq2 v1.42.0 R package [38], differentially abundant microbial taxa were identified using log2 fold‐change values and corresponding *p*‐values obtained from the Wald test. Taxa were selected based on the following criteria: (1) absolute log2 fold‐change > 0.3 and (2) *p*‐value < 0.01.

## Author Contributions

Conceptualization: J.K., Y.K., Y.J.L., E.S.J., and J.J. Methodology: J.K., Y.K., Y.J.L., H.I., H.K., C.S., S.J.K., Y.J.M., H.L., D.H.S., and E.S.J. Investigation: J.K., Y.K., Y.J.L., H.I., H.K., C.S., S.J.K., Y.J.M., H.L., D.H.S., and E.S.J. Visualization: J.K., H.I., H.K., C.S., S.J.K., and Y.J.M. Supervision: E.S.J. and J.J. Writing – original draft: J.K. and Y.K. Writing – review and editing: J.K., Y.K., E.S.J., and J.J. All authors have reviewed and approved the final version of the manuscript.

## Ethics Statement

Study approval was obtained from the Ulsan University Hospital Ethics Committee (March 21, 2022, No.: 2022‐01‐020), and written informed consent was obtained from all participants. This study was conducted in accordance with the principles of the Declaration of Helsinki.

## Conflicts of Interest

Hyo‐Jin Lee, Inseon Sim, Dong Ho Suh, and Eun Sung Jung are employees of HEM Pharma Inc. and participated in this work with non‐financial support. The remaining authors declare no conflicts of interest.

## Supporting information




**Supporting Figure 1**: Bacterial changes and metabolites alteration during HSCT. (A) Significantly shifted bacterial taxa by HSCT for whole HSCT period. (B) Bacterial taxa which altered with HSCT for T1 vs. T2, (C) T1 vs. T3, and (D) T2 vs. T3. (E) Heatmap of metabolic changes across HSCT stages. Metabolites were selected based on VIP > 0.7 and Kruskal‐Wallis H test (Bonferroni‐adjusted p‐value < 0.05). Abundance levels were Z‐score normalized, with red indicating increased and blue indicating decreased metabolite levels. Hierarchical clustering (Ward.D2) was applied to group metabolites with similar patterns, revealing distinct metabolic profiles across HSCT stages. (F) Volcano plots of differentially expressed metabolites across HSCT stages. These volcano plots show metabolite differences between T1 vs. T2 (left), T2 vs. T3 (middle), and T1 vs. T3 (right) based on log2 fold change (x‐axis) and ‐log10 adjusted p‐value (y‐axis). Red dots indicate significantly altered metabolites (p < 0.05, |log2FC| > 1), while gray dots represent non‐significant changes. The plots highlight key metabolic shifts across HSCT stages. (G‐I) Enrichment Analysis of Metabolic Pathways Across HSCT Stages. (G) T1‐T2, 39 metabolites, (H) T2‐T3, 12 metabolites, (I) T1‐T3, 53 metabolites. Enrichment analysis of T1 vs. T2 (39 metabolites), T2 vs. T3 (12 metabolites), and T1 vs. T3 (53 metabolites) based on the Wilcoxon signed‐rank test (Bonferroni adjusted) was performed using pathway information from the Small Molecule Pathway Database (SMPDB). The bar plot (left) ranks pathways by enrichment ratio, with colors indicating p‐value. The bubble plot (right) visualizes enrichment ratio (size) and significance (color). Key pathways, including glutathione, amino acid, and fatty acid metabolism, show significant shifts, reflecting metabolic adaptations during HSCT. Error bars represent the mean ± s.d. Source data are provided as a Source Data file.
**Supporting Figure 2**: Microbiota changes in response to GVHD and NF factors. (A) Relative abundance of meaningful changed specific bacteria for GVHD case. (B) Bacterial taxa which altered with GVHD for T1 vs. T2, (C) T1 vs. T3, and (D) T2 vs. T3. (E) Alpha‐diversity estimation of fecal microbiome for NF factors. Error bars represent the mean ± s.d. Source data are provided as a Source Data file.
**Supporting Figure 3**: Bacterial changes with HSCT complication G2 diarrhea. (A) Relative abundance of specific bacteria which showed notable changes for G2 diarrhea days. (B) Bacterial taxa which altered with G2 diarrhea factor for T1 vs. T2, (C) T1 vs. T3, and (D) T2 vs. T3. Error bars represent the mean ± s.d. Source data are provided as a Source Data file.
**Supporting Figure 4**: Metabolites changes with HSCT complications. Significant changes of metabolites concentration dependent on (A) NF, (B) G2 diarrhea, and (C) GVHD factors. Error bars represent the mean ± s.d. Source data are provided as a Source Data file.
**Supporting Figure 5**: Bacterial and metabolic changes during HSCT with transplantation types. **(A)** Alpha‐diversity measure of fecal microbiome for auto‐ (N = 35) and allo‐HSCT (N = 23) group. (B) Beta‐diversity analysis of time point‐dependent samples with divided by GVHD factor. (C) Bacterial taxa which altered with G2 diarrhea factor for T1 vs. T2, (D) T1 vs. T3, and (E) T2 vs. T3. Significant alteration of (F) SCFA and (G) specific metabolites for auto‐ versus allo‐HSCT patients. (H) Overall survival stratified by alpha‐diversity of transplantation type (p = 0.0028). Error bars represent the mean ± s.d. Source data are provided as a Source Data file.

## Data Availability

Data from this study will not be made publicly available to maintain participant confidentiality. Source data are provided with this paper. All raw DNA sequencing data have been deposited in the NCBI Sequence Read Archive under BioProject accession number PRJNA1234861. The metabolomics data have been deposited to MetaboLights repository with the study identifier MTBLS12365 (https://www.ebi.ac.uk/metabolights/reviewer25ccecd6‐59df‐423c‐ba77‐b11e0f9066c8).
