# Supplementary material for: Gut Microbiome and Metabolome Dynamics as Predictors of Clinical Outcomes in Hematopoietic Stem Cell Transplantation
Source: MedComm (2020). 2025 Aug 18;6(9):e70334. doi: 10.1002/mco2.70334 (PMC12360332; doi:10.1002/mco2.70334)
Supplement: Supplementary file 1 — Supporting Figure 1: Bacterial changes and metabolites alteration during HSCT. (A) Significantly shifted bacterial taxa by HSCT for whole HSCT period. (B) Bacterial taxa which altered with HSCT for T1 vs. T2, (C) T1 vs. T3, and (D) T2 vs. T3. (E) Heatmap of metabolic changes across HSCT stages. Metabolites were selected based on VIP > 0.7 and Kruskal‐Wallis H test (Bonferroni‐adjusted p‐value < 0.05). Abundance levels were Z‐score normalized, with red indicating increased and blue indicating decreased metabolite levels. Hierarchical clustering (Ward.D2) was applied to group metabolites with similar patterns, revealing distinct metabolic profiles across HSCT stages. (F) Volcano plots of differentially expressed metabolites across HSCT stages. These volcano plots show metabolite differences between T1 vs. T2 (left), T2 vs. T3 (middle), and T1 vs. T3 (right) based on log2 fold change (x‐axis) and ‐log10 adjusted p‐value (y‐axis). Red dots indicate significantly altered metabolites (p < 0.05, |log2FC| > 1), while gray dots represent non‐significant changes. The plots highlight key metabolic shifts across HSCT stages. (G‐I) Enrichment Analysis of Metabolic Pathways Across HSCT Stages. (G) T1‐T2, 39 metabolites, (H) T2‐T3, 12 metabolites, (I) T1‐T3, 53 metabolites. Enrichment analysis of T1 vs. T2 (39 metabolites), T2 vs. T3 (12 metabolites), and T1 vs. T3 (53 metabolites) based on the Wilcoxon signed‐rank test (Bonferroni adjusted) was performed using pathway information from the Small Molecule Pathway Database (SMPDB). The bar plot (left) ranks pathways by enrichment ratio, with colors indicating p‐value. The bubble plot (right) visualizes enrichment ratio (size) and significance (color). Key pathways, including glutathione, amino acid, and fatty acid metabolism, show significant shifts, reflecting metabolic adaptations during HSCT. Error bars represent the mean ± s.d. Source data are provided as a Source Data file. Supporting Figure 2: Microbiota changes in response to [file MCO2-6-e70334-s001.docx]

**Supplementary Information**

**Gut microbiome and metabolome dynamics as predictors of clinical outcomes in hematopoietic stem cell transplantation**

Juewon Kim1#, Youjin Kim^2^#, Yoo Jin Lee^2^, Hyo-Jin Lee^3^, Inseon Sim^3^, SuJin Koh^2^, Dong Ho Suh^3^, Eun Sung Jung^3*^, Jae-Cheol Jo^2*^

^1^Department of Physiology, Konkuk University College of Medicine, Chungju, Republic of Korea

^2^Department of Hematology and Oncology, Ulsan University Hospital, University of Ulsan

College of Medicine, Ulsan, Republic of Korea

^3^HEM Pharma Inc., Suwon, Republic of Korea

**Fig. S1-S5**

Fig S1. Bacterial changes and metabolites alteration during HSCT. (A) Significantly shifted bacterial taxa by HSCT for whole HSCT period. (B) Bacterial taxa which altered with HSCT for T1 vs. T2, (C) T1 vs. T3, and (D) T2 vs. T3. (E) Heatmap of metabolic changes across HSCT stages. Metabolites were selected based on VIP > 0.7 and Kruskal-Wallis H test (Bonferroni-adjusted p-value < 0.05). Abundance levels were Z-score normalized, with red indicating increased and blue indicating decreased metabolite levels. Hierarchical clustering (Ward.D2) was applied to group metabolites with similar patterns, revealing distinct metabolic profiles across HSCT stages. (F) Volcano plots of differentially expressed metabolites across HSCT stages. These volcano plots show metabolite differences between T1 vs. T2 (left), T2 vs. T3 (middle), and T1 vs. T3 (right) based on log2 fold change (x-axis) and -log10 adjusted p-value (y-axis). Red dots indicate significantly altered metabolites (p < 0.05, |log2FC| > 1), while gray dots represent non-significant changes. The plots highlight key metabolic shifts across HSCT stages. (G-I) Enrichment Analysis of Metabolic Pathways Across HSCT Stages. (G) T1-T2, 39 metabolites, (H) T2-T3, 12 metabolites, (I) T1-T3, 53 metabolites. Enrichment analysis of T1 vs. T2 (39 metabolites), T2 vs. T3 (12 metabolites), and T1 vs. T3 (53 metabolites) based on the Wilcoxon signed-rank test (Bonferroni adjusted) was performed using pathway information from the Small Molecule Pathway Database (SMPDB). The bar plot (left) ranks pathways by enrichment ratio, with colors indicating p-value. The bubble plot (right) visualizes enrichment ratio (size) and significance (color). Key pathways, including glutathione, amino acid, and fatty acid metabolism, show significant shifts, reflecting metabolic adaptations during HSCT. Error bars represent the mean ± s.d. Source data are provided as a Source Data file.

Fig S2. Microbiota changes in response to GVHD and NF factors. (A) Relative abundance of meaningful changed specific bacteria for GVHD case. (B) Bacterial taxa which altered with GVHD for T1 vs. T2, (C) T1 vs. T3, and (D) T2 vs. T3. (E) Alpha-diversity estimation of fecal microbiome for NF factors. Error bars represent the mean ± s.d. Source data are provided as a Source Data file.

Fig S3. Bacterial changes with HSCT complication G2 diarrhea. (A) Relative abundance of specific bacteria which showed notable changes for G2 diarrhea days. (B) Bacterial taxa which altered with G2 diarrhea factor for T1 vs. T2, (C) T1 vs. T3, and (D) T2 vs. T3. Error bars represent the mean ± s.d. Source data are provided as a Source Data file.

Fig S4. Metabolites changes with HSCT complications. Significant changes of metabolites concentration dependent on (A) NF, (B) G2 diarrhea, and (C) GVHD factors. Error bars represent the mean ± s.d. Source data are provided as a Source Data file.

Fig S5. Bacterial and metabolic changes during HSCT with transplantation types. **(A)** Alpha-diversity measure of fecal microbiome for auto- (N = 35) and allo-HSCT (N = 23) group. (B) Beta-diversity analysis of time point-dependent samples with divided by GVHD factor. (C) Bacterial taxa which altered with G2 diarrhea factor for T1 vs. T2, (D) T1 vs. T3, and (E) T2 vs. T3. Significant alteration of (F) SCFA and (G) specific metabolites for auto- versus allo-HSCT patients. (H) Overall survival stratified by alpha-diversity of transplantation type (p = 0.0028). Error bars represent the mean ± s.d. Source data are provided as a Source Data file.

Methods

Information of sequencing data
